# 13. The Efficacy and Effectiveness of Pneumococcal Vaccines against Pneumococcal Pneumonia among Adults: A Systematic Review and Meta-Analysis

**DOI:** 10.1093/ofid/ofab466.215

**Published:** 2021-12-04

**Authors:** Lana Childs, Miwako Kobayashi, Jennifer Loo Farrar, Tamara Pilishvili

**Affiliations:** 1 National Foundation for the Centers for Disease Control and Prevention, Inc., Atlanta, Georgia; 2 Centers for Disease Control and Prevention, Atlanta, GA; 3 Centers for Disease Control and Prevention, Atlanta, GA, USA, Atlanta, Georgia

## Abstract

**Background:**

Two pneumococcal vaccines are currently recommended for use in U.S. adults: 23-valent pneumococcal polysaccharide vaccine (PPSV23) and 13-valent pneumococcal conjugate vaccine (PCV13). Recommendations for adult PCV13 use were supported by a large randomized-controlled trial (RCT) demonstrating PCV13 efficacy against pneumococcal pneumonia (PnPn) and vaccine-type (VT) PnPn in older adults. New pneumococcal conjugate vaccines are expected to be licensed for adults in late 2021 and recommendations for use among adults will be reviewed and revised, as needed. We conducted a systematic review to summarize evidence on the vaccine efficacy and effectiveness (VE) of PPSV23 and PCV13 against PnPn among adults.

**Methods:**

We conducted a search of literature published from 1998 to February 2021 on PCV13 and PPSV23 VE studies using eight reference databases. Studies targeting adults with immunocompromising conditions were excluded. VE results with 95% confidence intervals (CI) were abstracted and stratified by vaccine product, outcome evaluated (PnPn and VT PnPn), study design, and effect measure. When applicable, random effects models were used to estimate pooled VE and I-squared statistic was reported to assess heterogeneity.

**Results:**

Of 3,422 screened studies, we included 15 studies: three on PCV13 and 12 on PPSV23 (Table 1). In addition to the RCT, we identified two observational studies for PCV13 (Table 1); however, pooled VE of the observational studies was not estimated due to differences in methods for reporting results. Pooled PPSV23 VE against PnPn from two RCTs was 63% (95% CI: 31, 80 I^2^=0%). Pooled VE of PPSV23 against VT PnPn from three observational studies was 18% (95% CI: -35, 35 I^2^=38%). PPSV23 effectiveness against PnPn was limited with a pooled VE of 25% (95% CI: 7, 39 I^2^=78%) from nine observational studies.

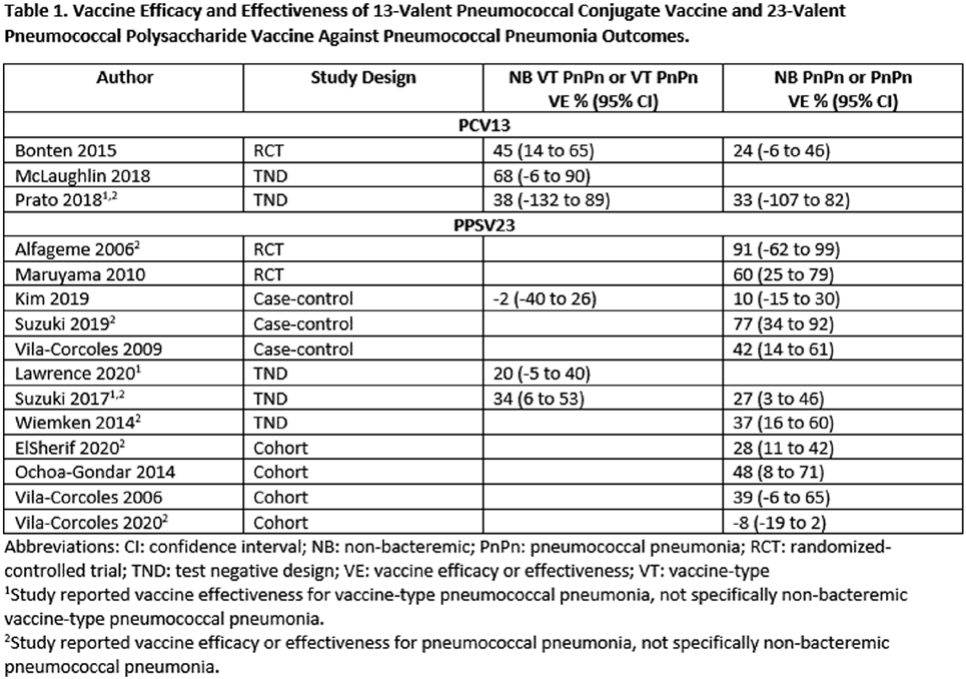

**Conclusion:**

Findings from observational studies supported PCV13 VE against VT PnPn reported in the RCT. Differences in the study design made the magnitude of PPSV23 effectiveness against PnPn and VT PnPn difficult to assess; however, findings from recent observational studies suggest PPSV23 provides limited protection against VT PnPn.

**Disclosures:**

**All Authors**: No reported disclosures

